# Comparative Study of the Detection of Chromium Content in Rice Leaves by 532 nm and 1064 nm Laser-Induced Breakdown Spectroscopy

**DOI:** 10.3390/s18020621

**Published:** 2018-02-18

**Authors:** Jiyu Peng, Fei Liu, Tingting Shen, Lanhan Ye, Wenwen Kong, Wei Wang, Xiaodan Liu, Yong He

**Affiliations:** 1 College of Biosystems Engineering and Food science, Zhejiang University, Hangzhou 310058, China; jypeng@zju.edu.cn (J.P.); fliu@zju.edu.cn (F.L.); shentingtingstt@163.com (T.S.); lena_ye15@163.com (L.Y.); 15236193955@163.com (W.W.); m15307266704@163.com (X.L.); 2 Key Laboratory of Spectroscopy Sensing, Ministry of Agriculture, Hangzhou 310058, China; 3 School of Information Engineering, Zhejiang A & F University, Linan 311300, China; zjukww@163.com

**Keywords:** rice leaves, chromium content, laser-induced breakdown spectroscopy, laser wavelength, preprocessing methods

## Abstract

Fast detection of toxic metals in crops is important for monitoring pollution and ensuring food safety. In this study, laser-induced breakdown spectroscopy (LIBS) was used to detect the chromium content in rice leaves. We investigated the influence of laser wavelength (532 nm and 1064 nm excitation), along with the variations of delay time, pulse energy, and lens-to-sample distance (LTSD), on the signal (sensitivity and stability) and plasma features (temperature and electron density). With the optimized experimental parameters, univariate analysis was used for quantifying the chromium content, and several preprocessing methods (including background normalization, area normalization, multiplicative scatter correction (MSC) transformation and standardized normal variate (SNV) transformation were used to further improve the analytical performance. The results indicated that 532 nm excitation showed better sensitivity than 1064 nm excitation, with a detection limit around two times lower. However, the prediction accuracy for both excitation wavelengths was similar. The best result, with a correlation coefficient of 0.9849, root-mean-square error of 3.89 mg/kg and detection limit of 2.72 mg/kg, was obtained using the SNV transformed signal (Cr I 425.43 nm) induced by 532 nm excitation. The results indicate the inspiring capability of LIBS for toxic metals detection in plant materials.

## 1. Introduction

Toxic metal detection is important for monitoring environmental pollution and ensuring food safety, which is directly related to human’s health. As the second most common metal contaminant in soil, water and sediments, chromium (Cr) can cause severe morphological, physiological and biochemical process damage in plants [1]. Because of the advantages of fast analytical speed, non-contact detection and little sample preparation, the laser-induced breakdown spectroscopy (LIBS) technique has been proven efficient in the determination of elements in biological samples, such as plant materials [2,3], algal biomass [4], and biomedical samples [5].

Several studies concerning the detection of chromium [6,7], copper [6,8], lead [9] and silver [10] contents in plant materials have been published recently. However, due to the moderate detection limits of LIBS, the samples used for analysis usually had a high toxic metal contents [9], and the sample number was limited [6], making it difficult to evaluate the quantification capability of the LIBS technique. On the other hand, the application of LIBS in toxic metals detection is also limited by the instability of signal, which is mainly dependent of the sampling approach (laser ablation), sampling mass (usually around μg/pulse) and complexity of the laser-sample interaction [11]. The very characteristics of LIBS might be considered as some of the most important issues for quantification of toxic metals in plant materials.

In general, the analytical performance of LIBS could be improved by choosing suitable experimental parameters (e.g., laser properties, beam guiding strategies and detector parameters). The laser wavelength is one of important parameters that is related to the laser-sample and laser-plasma interactions, which might further affect the LIBS signal. The influence of laser wavelength on the absorption properties of substance and plasma has been discussed by Noll et al. in his book [12]. It has been also reported that shorter wavelengths had higher photon energies that might lead to shorter optical penetration depth, higher ablation rate and less fractionation [13]. In contrast, longer wavelengths might have stronger plasma shielding and inverse bremsstrahlung [14]. Stronger emissions and weaker signal stability were observed in IR irradiation [15]. Zhang et al. compared the signal intensity and stability under 266 nm and 1064 nm excitations when analyzing trace elements in holly leaves [16]. Better repeatability was found under 266 nm excitation. The Nd:YAG laser operated at its fundamental wavelength (1064 nm) is mostly preferred by researchers because of its economic and powerful features. In this case, some efforts are carried out to evaluate the influence of laser wavelength.

In addition to the laser wavelength, the analytical performance of LIBS is also greatly affected by laser energy, lens-to-sample distance (LTSD) and delay time [12]. All these factors along with laser wavelength determine the overall performance of LIBS. De Carvalho et al. investigated the effects of fluence on the detection of nutrient elements in plant materials using LIBS. They found that the sensitivity and measurement precision increased with laser fluence [17]. In addition, the gate width, delay time, amplification gain and number of pulses for detection of nutrient elements in plants materials were optimized based on a neuro-genetic approach [18]. However, few studies have considered the influence of wavelength as well as the correlation with other factors, especially for plant materials. Different from other samples (e.g., metals), the constituents of plant materials are complex, inhomogeneous, and it may be sensitive to the experimental parameters.

From the point of view of modeling, data preprocessing is necessary for LIBS data management. Because of the laser ablation process in LIBS, the accuracy and precision of analytical results might be affected by random or systematic changes of the abovementioned parameters [19]. It has been proven that normalization and outliers filtering could be used to reduce the interference matrix and to improve the calibration models [20,21,22]. For the normalization strategies, it mainly consists of single-variate and multivariate corrections, which has been reviewed by Zorov et al. in detail [23]. A single reference signal (e.g., individual line, background, spectral area) is used in single-variate corrections, while more than one reference signal is used in multivariate corrections. In this study, a self-developed routine was used to detect outliers and four different normalization methods were used to reduce shot-to-shot fluctuation.

In this work, we aimed to detect chromium content in rice leave using fundamental wavelength (1064 nm, *E_p_* = 1.17 eV) and second harmonic (532 nm, *E_p_* = 2.34 eV) of Nd:YAG Q-switched pulse laser. The specific objectives of this study were (1) to compare the influence of wavelength on signal (sensitivity and repeatability), plasma features (electron density and temperature) and quantification capability; (2) to optimize the experimental parameters for both 532 nm and 1064 nm laser wavelengths; (3) to establish univariate models for the fast detection of chromium content in rice leaves, and further improve the performance with preprocessing methods (background normalization, area normalization, standardized normal variate (SNV) and multiplicative scatter correction (MSC)).

## 2. Materials and Methods

### 2.1. Sample Preparation

Rice (*Oryza sativa* L.) leaves with different chromium stress were used for analysis. Rice seeds with genotype Chunyou 84 were obtained from the China National Rice Research Institute (Hangzhou, China). The cultivation of rice plants was carried out according to Zeng et al. [24], and the nutrient solution was prepared according to Yoshida et al. [25]. After growing in complete nutrient solution for 7 days, five treatments including control group and experiment groups of 25 μM, 50 μM, 75 μM and 100 μM chromium stress (prepared using K_2_Cr_2_O_7_) were adopted. The chromium stress of 50 μM and 100 μM could be considered as moderate pollution and severe pollution, respectively [26]. In addition, an extra group with a chromium stress of 60 μM was prepared to investigate the influence of wavelength on the variations of delay time, pulse energy and LTSD. After exposure to chromium stress for 8 weeks, 43 samples with the five treatments (see Table 1) were collected, and all rice leaves of the extra group were used to constitute one sample. In order to reduce shot-to-shot fluctuation, all the samples were dried, ground and pressed into pellets. The leaves were ground with a tissue milling machine. The samples were placed in a 5 mL centrifuge tube, and ground at 60 Hz for 1 min. The pellets were prepared by placing 100 mg of leaf powder into a squared die set and then pressed with 600 MPa of pressure for 1 min. The pellets were square with 10 mm length on each side.

### 2.2. Experimental Setup

The experiment was carried out with a self-assembled LIBS setup, which mainly consists of a Nd:YAG Q-switched pulse laser for ablation, optics for beam guiding, spectrograph for light dispersion and intensified charge coupled device (ICCD) camera for detection. A detailed description of our LIBS device has been given elsewhere [27].

In this work, 532 nm and 1064 nm excitations were produced when laser operated at the fundamental and second harmonics, respectively. The beam profile of the laser was nearly a flat top distribution. The beam diameter and pulse duration were 7 mm and 8 ns, respectively. In addition, the RSD of pulse-to-pulse energy was less than 2%. With the help of a plano-convex lens (f = 100 mm, 532/1064 nm V coat), the laser beams were focused onto samples. In order to avoid ablating the same spot continuously, the samples were moved by an X-Y-Z motorized stage every five accumulation shots, and a total of 16 positions were ablated for each sample. The relative gain of ICCD was set at 26. Before the experiment, wavelength of spectrograph and signal intensity of ICCD were calibrated using a mercury argon lamp (HG-1, Ocean Optics, Minneola, FL, USA) and a deuterium halogen source (DH-2000-BAL-CAL, Ocean Optics, Minneola, FL, USA).

### 2.3. Reference Method for Chromium Content Determination

The reference value of chromium content of rice leaves was determined with inductively coupled plasma mass spectrometry (ICP-MS), using a modification of method described by Aziz et al. [28]. In addition, the trueness of the reference method was evaluated with citrus leaves (GBW10020), and the result was consistent with the certified value. Before the measurement, 100 mg of ground leaves was weighted into the Teflon vessels and microwave digested with 4 mL of 65% HNO_3_ and 1 mL of 30% H_2_O_2_. After digestion, they were translated into 25 mL volumetric flasks and diluted to the mark with distilled water. Finally, the solutions were analyzed with ICP-MS (ELAN DRC-e, PerkinElmer, Waltham, MA, USA). The duration of sample preparation (regardless of drying and grinding) for ICP-MS was about 3 h, and it was 2 min for LIBS. The results for chromium content determination were shown in Table 1.

### 2.4. Data Analysis

In LIBS analysis, abnormal spectra might result due to the fluctuation of experimental parameters. It has been reported that removing the outliers in data analysis could improve the precision and repeatability of LIBS measurement [29]. After LIBS data acquisition, a self-developed routine was used to remove abnormal spectra based on median absolute deviation (MAD) [30]. In order to meet our particular case, we used the peak intensity of emission CN 388.29 nm as a variable to identify outliers. Because the emission of molecule band CN usually appeared in organic samples, and it was relatively stable. First, the median and MAD of peak intensity of CN 388.29 nm was calculated. Then the spectrum was considered as an outlier when the difference between its intensity of CN 388.29 nm and median was beyond 2.5 times MAD. Outlier detection was performed until no outlier was identified or the number of remaining spectra was less than 75% of the number of original spectra. In order to obtain the real response of the system, the process of outliers filtering was not applied in optimization, while it was only performed during the modeling.

In order to compare the signal quality and performance metrics of LIBS with different experimental parameters, we used limit of detection (LOD), signal-to-noise (S/N) and signal-to-background (S/B) ratios to evaluate sensitivity and relative standard deviation (RSD) to evaluate signal stability. These indicators were calculated with following equations:

(1) $$\frac{S}{N}=\frac{I_{net\;signal}}{\sigma_{background}}$$

(2) $$\frac{S}{B}=\frac{I_{net\;signal}}{{\bar{I}}_{background}}$$

(3) $$\mathrm{RSD}=\frac{\sigma_{signal}}{{\bar{I}}_{signal}}\times 100\%$$

where *I_net signal_* is the net signal of interested element, *σ_background_* is the standard deviation of the background intensities, $\bar{I}$*_background_* is the averaged intensity of background near to analytical line, *σ_signal_* is standard deviation of signal of interested element with three replicate measurements, and $\bar{I}$*_signal_* is averaged intensity of signal of interested element with three replicate measurements.

In addition, electron density and temperature were used to characterize the plasma features. Because the electron density was related to the full width at half maximum (FWHM) of the Stark broadening lines, it could be calculated with following equation:

(4) $$\Delta \lambda_{stark}=2\omega \left[\frac{N_{e}}{10^{16}}\right]$$

where $N_{e}$ is the electron density (cm^−3^), ω is the electron impact width parameter [31], and $\Delta \lambda_{stark}$ is FWHM of Stark broadening lines (i.e., Mg II 280.27 nm in this case, and no self-absorption and overlapping were observed). Because the measured line broadening could be dominated by Stark broadening and instrumental broadening, we used a Voigt function to fit the experimental data. The term $\Delta \lambda_{stark}$ could be deduced with following expression [32]:

(5) $$\Delta \lambda_{exp}=\frac{\Delta \lambda_{stark}}{2}+{\left[{\left(\frac{\Delta \lambda_{stark}}{2}\right)}^{2}+{\left(\Delta \lambda_{inst}\right)}^{2}\right]}^{1/2}$$

where $\Delta \lambda_{exp}$ is the FWHM of experimental broadening of Mg II 280.27 nm, and $\Delta \lambda_{inst}$ is the instrumental broadening of the spectrograph, which was measured using a mercury light source. In this case, $\Delta \lambda_{inst}$ was determined to be 0.055 nm (in air).

The temperature of plasma was estimated by spectrum simulation of the CN emissions in the spectral window around 388 nm using LIFBASE2.1 software [33].

After investigating the influences and optimizing the values of experimental parameters, univariate analysis was used to compare the quantification performance with 532/1064 nm excitations. The order of samples (*n* = 43) was rearranged according to the chromium content from low to high. Then, three samples with an interval of four were assigned to a calibration set (*n* = 32), and the rest were in the prediction set (*n* = 11).

In addition, normalization was used to eliminate the matrix effect and shot-to-shot fluctuation and further improve the calibration performance [23]. In this case, different prepocessing methods were compared, including background normalization, area normalization, SNV and MSC. MSC is a transformation method that is originally used to compensate for additive and multiplicate scattering in reflectance spectroscopy [34]. It consists of fitting a separate regression line to each spectrum using mean spectrum of a set of samples, and the coefficients are used to correct the variables in original spectrum. SNV is another transformation method that is also used for correcting multiplicate scattering in spectroscopy [35]. Different from MSC, it removes the scatter effect by centering and scaling each spectrum only with data from that spectrum. Background and area normalizations were obtained by normalizing each spectrum to background intensity and the total emission integrated intensity, respectively. SNV and MSC transformations were performed in the Unscrambler X (CAMO AS, Oslo, Norway).

The calibration results were evaluated with correlation coefficient (R) and root of mean squared error (RMSE). RMSE is a measurement of the average difference between reference values and model predicted values. It is obtained by calculating the square root of the average of squared errors. The sensitivity of proposed methods was compared using LOD, which could be deduced with following equation:

(6) $$\mathrm{LOD}=\frac{3\sigma_{background}}{b}$$

where *σ_background_* is the standard deviation of the background intensities, *b* is the slope of calibration curve.

## 3. Results

### 3.1. Spectral Characteristics

**Figure 1:**
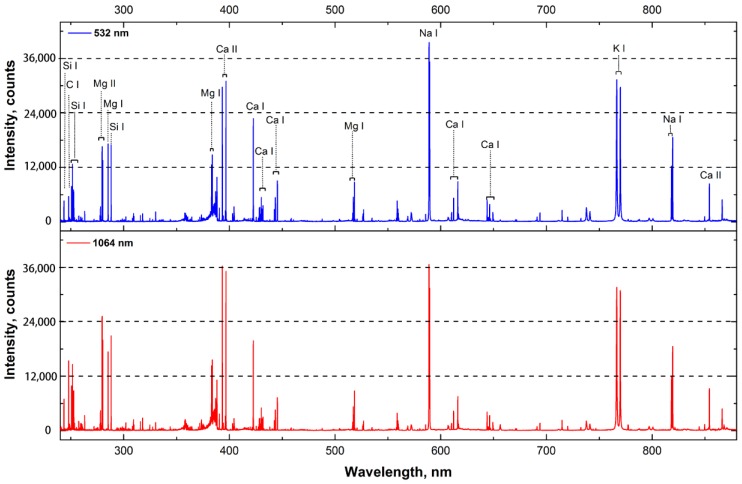
Averaged spectra of sample from extra group (60 μM chromium stress) in the range of 240–880 nm. Experimental parameters of energy, LTSD, delay time, gate width for 532 nm and 1064 nm excitations are 90 mJ, 98 mm, 4 μs and 16 μs, respectively.

Figure 1 shows the averaged spectra of sample from extra group (60 μM chromium stress) with 532/1064 nm excitations. In order to investigate the influence of laser wavelength, the experimental parameters of pulse energy, LTSD, delay time and gate width were set at 90 mJ, 98 mm, 4 μs and 16 μs, respectively. As seen in Figure 1, the emission lines from macro-nutrients (such as Ca, Mg, K, Si) and micro-nutrients (Na) were observed, as well as the main organic constituent (C). Distinguishing differences in line intensity were observed for 532/1064 nm excitations, and the differences were associated with the wavelength of emission lines. In the spectrum near UV region, the intensity with 1064 nm excitation was more intense than that from 532 nm excitation. However, in the visible and near-IR region, the intensity of most emission lines with 532 nm excitation was higher than that from 1064 nm excitation. This might be related to the transition energy of emission lines. According to the equation ΔE=hc/λ, the transition energy was inversely proportional to the wavelength. Hence, the emission line with longer wavelength corresponded to lower transition energy. Because photon energy of 532 nm excitation (*E_p_* = 2.34 eV) was higher than 1064 nm excitation (*E_p_* = 1.17 eV), the processes of bonding breaking and ionization were more intense [36]. Therefore, more transitions with 532 nm excitation might occur for lower energy gap (i.e., longer wavelength region).

For the emission lines of chromium (Cr I 425.43, 428.48 nm), the intensities with 532/1064 nm excitations in this experimental condition were similar. This was accordant with above conclusion that the spectral intensity was affected both by transition energy and inverse bremsstrahlung. As seen in Table 2, the transition energies for Cr I 425.43 nm and Cr I 428.48 nm were 2.91 eV and 2.90 eV, respectively. However, the prominent mechanism of the excitation wavelength differed with different experimental conditions (delay time, laser energy and LTSD). In order to find the best experimental parameters, we further investigated the influence of wavelength under above mentioned experimental parameters.

### 3.2. Influence of Delay Time

Delay time is an important parameter in LIBS detection, which describes the delay from initiation of plasma to detection. In the first period of plasma radiation process, the background continuum emission is strong, and it is mainly due to bremsstrahlung process. In this case, time-resolved analysis of plasma light allows for the determination of the suitable delay when the signal from interested emissions predominate. In addition, gate width allows for recording time period of the interested emissions, which is also called as integral time. In the pre-experiment, the life time of emission lines (Cr I 425.43, 427.48 nm) was estimated around 20 μs. In order to capture all the signal during the life time and eliminate the influence of dark current, the sum of delay time and gate width was equal to 20 μs. In addition, the laser energy and LTSD for both 532/1064 nm excitations were set at 80 mJ and 98 mm, respectively.

**Figure 2:**
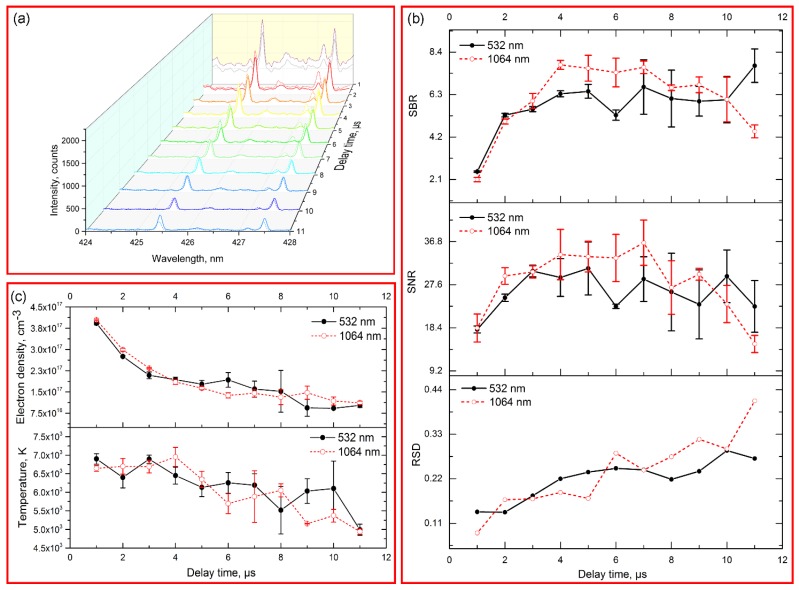
(**a**) Spectral intensity in the range of 424–428 nm with different delay times. The solid line indicated the spectra from 532 nm excitation, and dash line indicated the spectra from 1064 nm excitation; (**b**) Comparison of SBR, SNR and RSD of emission Cr I 425.43 nm with different delay time under 532 nm and 1064 nm excitations; (**c**) Time-resolved analysis of electron density and temperature under 532 nm and 1064 nm excitations.

Figure 2a shows the time-resolved spectral intensity in the range of 424–428 nm. In the early delay time, background continuum emissions were strong for both 532/1064 nm excitations, which resulted in low SNR/SBR for interested signal (see Figure 2b). As the increase of delay time, the background continuum emissions decreased quickly, while the intensity of emissions Cr I 425.43, 427.48 nm decreased slowly during the delay time of 0–4 μs. However, the RSD of interested signal continually increased as the increase of delay time, which indicated the decay of signal stability. The trend of RSD was contrast with those of temperature and electron density (Figure 2b,c). At long delay times, the plasma began to cool down, which caused the signal instability.

Some differences were observed for 532/1064 nm excitations. Compared with 532 nm excitation, both background continuum and analytical emissions for 1064 nm excitation were strong in the first delay period, while those decayed quickly as the increase of delay time. It was known that the first stage of plasma was predominated by inverse bremsstrahlung (free-free transition), whose cross-section was proportional to the cube of laser excitation wavelength [15]. Hence, higher absorption was observed in 1064 nm wavelength laser induced plasma. In the long delay time (≥10 μs), the emissions from 1064 nm excitation were weaker than those from 532 nm excitation. It indicated that the emissions from 1064 nm excitation might decay quickly than that from 532 nm excitation. Take overall consideration of SBR, SNR and RSD, the optimal delay time for 532/1064 nm excitations were both 4 μs.

### 3.3. Influence of Pulse Energy

Laser pulse energy is an important laser related parameter, which affects the ablation and plasma formation [37]. More precisely, the pulse energy used for ablation can be transformed to fluence (energy per unit area, J/cm^2^), which can better describe the energy delivered to the target. In this experiment, the energy was adjustable in the range of 20–130 mJ with an attenuator of half wave plate and polarizer, and the LTSD was maintained at 98 mm (i.e., the laser beam focused 2 mm below the target). Hence, the fluence used for ablation was in the range of 5.1–33.8 J/cm^2^ with interval of 2.55 J/cm^2^. In addition, the delay time and gate width of ICCD were optimized at 4 μs and 16 μs according to the results obtained from above section.

**Figure 3:**
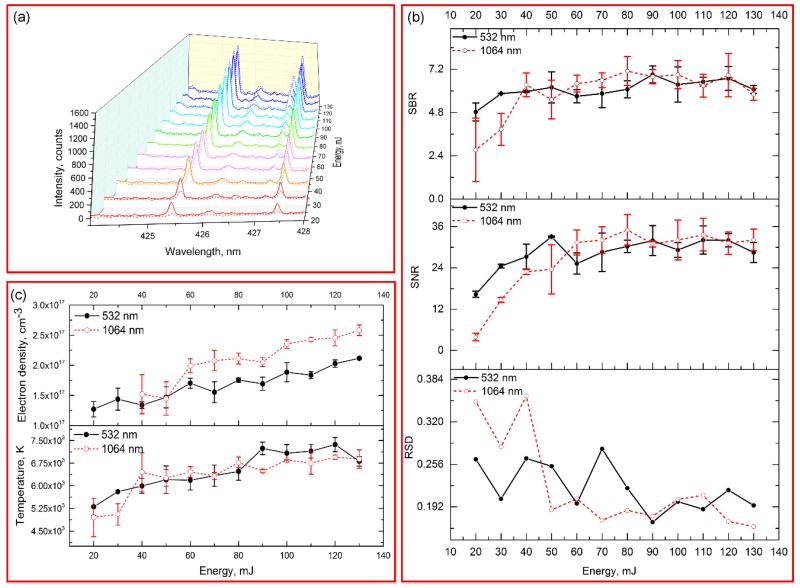
(**a**) Spectral intensity in the range of 424–428 nm with different laser energies. The solid line indicated the spectra from 532 nm excitation, and dash line indicated the spectra from 1064 nm excitation; (**b**) Comparison of SBR, SNR and RSD of emission Cr I 425.43 nm with different laser energies under 532 nm and 1064 nm excitations; (**c**) Electron density and temperature with different laser energy under 532 nm and 1064 nm excitations.

**Figure 4:**
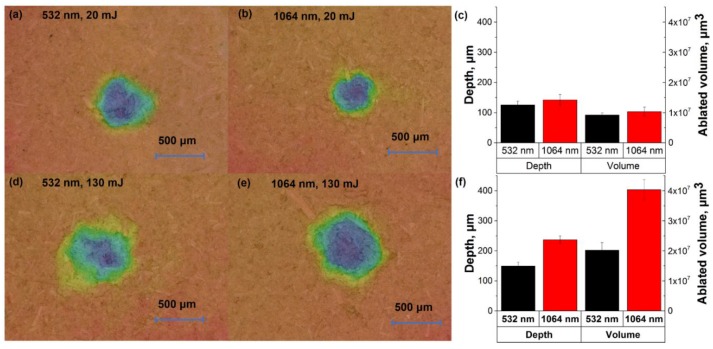
Morphologies of craters with different pulse energies under 532 nm and 1064 nm excitations: (**a**) 532 nm excitation, pulse energy = 20 mJ; (**b**) 1064 nm excitation, pulse energy = 20 mJ; (**d**) 532 nm excitation, pulse energy = 130 mJ; (**e**) 1064 nm excitation, pulse energy = 130 mJ. The colors in (**a,b,d,e**) indicated the depth of craters. Plots of statistic parameters (depth and ablated volume) of craters with different pulse energies: (**c**) pulse energy = 20 mJ; (**f**) pulse energy = 130 mJ. The morphologies of craters were measured using digital microscopy (VHX-6000, Keyence, Osaka, Japan).

Figure 3a shows the spectral intensity in the range of 424–428 nm with 12 different pulse energies of the laser (532/1064 nm, 8 ns, 1 Hz). The signal intensities for chromium increased with the increase of energy, while they began to flatten in high energy. The variation of background continuum emission was similar to the signal for chromium. The increase of spectral intensity might be related to the greater ablated particle with the increase of fluence (see Figure 4). Figure 3c shows the electron density and plasma temperature with different pulse energies. The electron density in low pulse energy (≤30 mJ) of 1064 nm excitation was not available, because the emission line used for calculation was disappeared. The electron density and temperature of plasma increased as the increase of energy (see Figure 3c). It indicated that more electrons were generated, and greater energy of photons were absorbed by electrons, which caused the increase of temperature [38]. In addition, when the fluence was low, the RSD were relatively high (Figure 3b). As the fluence used for the samples was around the ablation threshold, the produced plasma was likely unstable.

The differences of 532/1064 nm excitations with different pulse energies were also compared. In the low pulse energy, especially for the energy lower than 40 mJ, the signal intensity, SBR and SNR from 532 nm excitation were obviously higher than those from 1064 nm excitation (see Figure 3a,b). This phenomenon might be related to the actual energy for ablation in low pulse energy. Because of the plasma shielding effect, greater pulse energy of 1064 nm excitation was absorbed by the plasma, causing less energy for ablation [39]. As the increase of energy, the increase of ablation efficiency of 1064 nm excitation was obvious. As seen in Figure 4, the depth and ablated volume of crater from 1064 nm excitation increased greatly compared with those from 532 nm excitation. Thus, higher emissions were induced by 1064 nm excitation with high pulse energy. However, the SNR and SBR of 532/1064 nm excitations were similar when the energy higher than 80 mJ. For practical applications, using 532 nm excitation or 1064 nm excitation for element detection was similar when high pulse energy was required, while 532 nm excitation was suitable for low destruction applications, such as biological samples. The optimized energy for 532 nm and 1064 nm excitations in this case were 90 mJ and 80 mJ, of which the SBR/SNR reached the maximal and the RSD was relatively low.

### 3.4. Influence of Lens-To-Sample Distance

We also investigated the influence of lens-to-sample distance on the signal. LTSD not only affects the fluence delivered to the target, but also has an effect on signal stability. In fact, the LTSD is a brief description of the laser spot on the target with fixed experimental parameters, which is dependent of the focal length of lens and the diameter of laser beam. In this experiment, the focal length of lens was 100 mm, and the diameter of laser beam was around 7 mm. In addition, the delay time and gate width of camera were set at 4 μs and 16 μs, and the pulse energy used in this case was 80 mJ.

**Figure 5:**
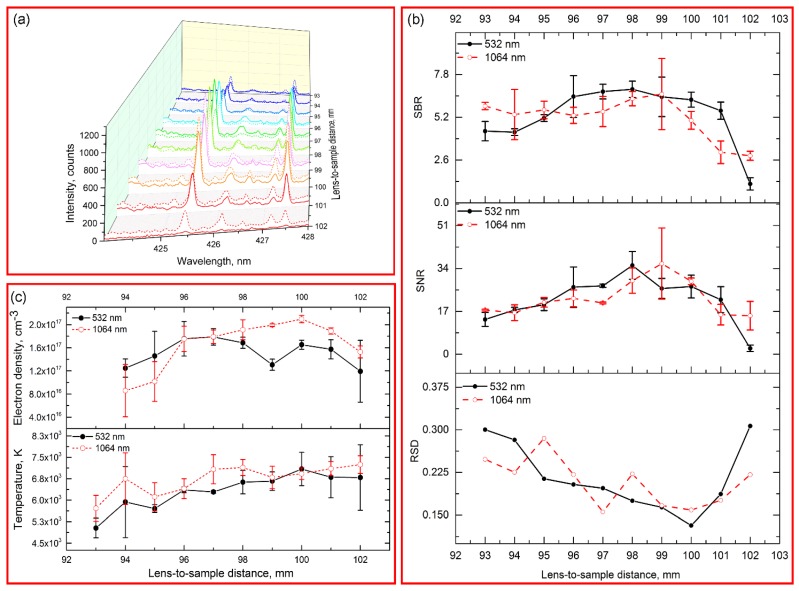
(**a**) Spectral intensity in the range of 424–428 nm with different LTSDs. The solid line indicated the spectra from 532 nm excitation, and dash line indicated the spectra from 1064 nm excitation; (**b**) Comparison of SBR, SNR and RSD of emission Cr I 425.43 nm with different LTSDs under 532 nm and 1064 nm excitations; (**c**) electron density and temperature with different LTSDs under 532 nm and 1064 nm excitations.

**Figure 6:**
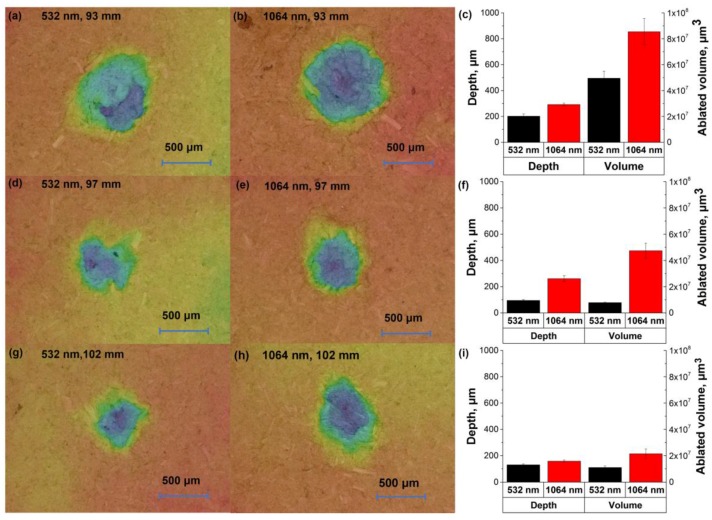
Morphologies of craters with different LTSDs under 532 nm and 1064 nm excitations: (**a**) 532 nm excitation, LTSD = 93 mm; (**b**) 1064 nm excitation, LTSD = 93 mm; (**d**) 532 nm excitation, LTSD = 97 mm; (**e**) 1064 nm excitation, LTSD = 97 mm; (**g**) 532 nm excitation, LTSD = 102 mm; (**h**) 1064 nm excitation, LTSD = 102 mm. The colors in (**a,b,d,e,g,h**) indicated the depth of craters. Plots of statistic parameters (depth and ablated volume) of craters with different LTSDs: (**c**) LTSD = 93 mm; (**f**) LTSD = 97 mm; (**i**) LTSD = 102 mm.

Figure 5a shows the spectral intensity in 424–428 nm spectral window with LTSD in the range of 93–102 mm. When the LTSD was larger than 100 mm, the laser ablated the atmosphere. As the increase of LTSD, the signal achieved its maximal values at 98 mm and 99 mm for 532/1064 nm excitations and then decreased. The emissions of chromium might not be directly related with crater’s depth and ablated volume. As seen in Figure 6, the ablated volume and depth decrease, while the emissions increase with the LTSD changes from 93 to 98 mm. Hence, the variance of emission intensity might be credited to the ablation efficiency between laser beam and target, which was related to ablation fluence and ablation spot. As the LTSD increased (<100 mm), the ablation fluence increased while the ablation spot decreased. The maximal values of signal might indicate the optimized values of LTSD with the largest ablation efficiency, which were based on the trade-off between the fluence and the ablation spot diameter. The trends of SNR and SBR were consistent with that of signal (Figure 5b). For the RSD, it deceased with LTSD from 93 to 100 mm, then it greatly increased. As seen in Figure 5c, the temperature and electron density increased with the increase of LTSD (when it was shorter than 100 mm). It indicated that the used pulse energy with short LTSD might be insufficient to produce enough electrons and maintain high plasma temperature, which induced instability of the signal. For the LTSD larger than the focal length of lens, the laser beam mainly caused the breakdown in the air rather than the samples. In the practical application, it should be strictly avoided. Above all, the optimal LTSD for 532 nm and 1064 nm excitations were 98 mm and 99 mm, respectively.

We also compared the influence of wavelength with the variation of LTSD. When the LTSD was in the range of 95 mm to 98 mm, the signal intensity and background continuum emission from 532 nm excitation was higher than those from 1064 nm excitation. It was contrary for the remaining values of LTSDs. The phenomenon might be credited to the greater ablation efficiency of 532 nm excitation and plasma shielding effect of 1064 nm excitation. As seen in Figure 6, the craters caused by 532 nm laser beam were apparently with smaller depth and ablated volume, and it was more obvious for 98 mm LTSD. For the LTSD larger than the focal length of lens, although similar depth and ablated volume were found with 532/1064 nm excitations, the emissions from 1064 nm excitation were apparently more intense than that from 532 nm excitation. Thanks to the greater optical penetration depth of 1064 nm excitation, more mass of sample was evaporated and transferred into plasma, which might emit intense chromium radiation. The differences between 532 and 1064 nm excitations could be credited to the different ablation efficiency and plasma shielding effect, which might be related to the ablation fluence and ablation spot. Hence, further study is necessary to improve the ablation efficiency and reduce the effect of plasma shielding by optimizing the experimental parameters.

### 3.5. Analytical Figures-Of-Merit

**Figure 7:**
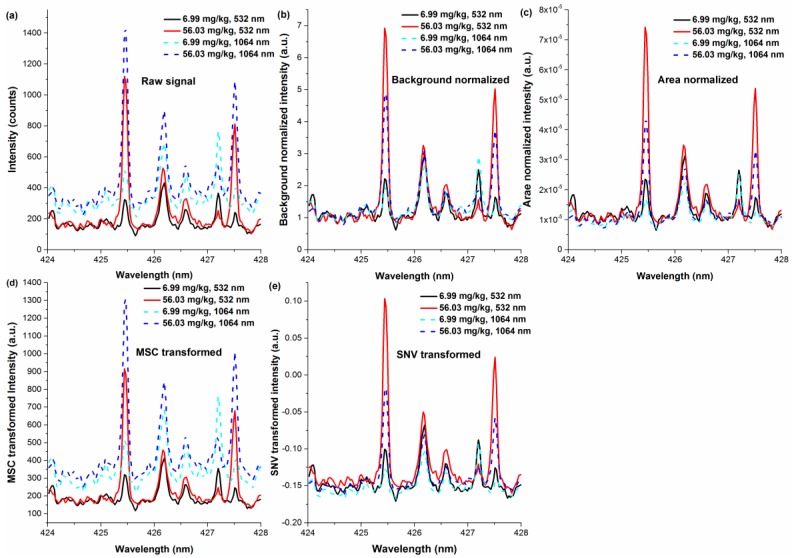
Spectra of two representative samples (chromium content of 6.99 mg/kg and 56.03 mg/kg) in the spectral range of 424–248 nm. The spectra were processed by different pretreatments: (**a**) raw; (**b**) background normalization; (**c**) area normalization; (**d**) MSC transformation; (**e**) SNV transformation.

After optimization of the experimental parameters under 532/1064 nm excitations, we further investigated the influence of wavelength on the calibration results. Pulse energy, delay time, gate width, LTSD for 532/1064 nm excitations were set at 90/80 mJ, 4/4 μs, 16/16 μs and 98/99 mm, respectively. Figure 7a shows the spectra of two representative samples with chromium content of 6.99 mg/kg and 56.03 mg/kg, respectively. After parameter optimization, the chromium signal and background continuum emission induced by 1064 nm excitation were both stronger than those from 532 nm excitation. However, it was hard to evaluate the influence of wavelength on the detection simply from two samples.

Figure 7b–e show the spectra after the treatments of background normalization, area normalization, MSC transformation and SNV transformation, respectively. As seen in Figure 7, the baselines of the spectra with background normalization, area normalization and SNV transformation were similar, while the baseline of MSC transformed and raw spectra show distinguished difference between 532 and 1064 nm excitations. This might be credited to the normalization principal of each method. Because MSC standardized each spectrum using the mean spectrum of each set as reference, the corrected spectra might retain basic features of experimental conditions and sample characteristics. Hence, the MSC normalization might be more suitable for similar sample sets, and the experimental conditions should be similar. This very feature might limit the practical application. In addition, the preprocessed spectra of background normalization, area normalization and SNV normalization showed similar trend, and the chromium emissions from 532 nm excitation were greatly stronger than that from 1064 nm excitation. As seen in Figure 7a, the raw chromium signal of 532/1064 nm excitations was similar. Hence, the decreased chromium signal in preprocessed spectra might be attributed to the variables used for normalization. It also indicated that 1064 nm excitation might induce greater background, integral area and noise.

We further compared the calibration results with different preprocessing methods in Table 3. The analytical signal used for calibration was the maximal value within three pixels around Cr I at 425.43 nm and 427.48 nm. The model performance was evaluated with RMSE and R, and the sensitivity was evaluated with LOD.

**Figure 8:**
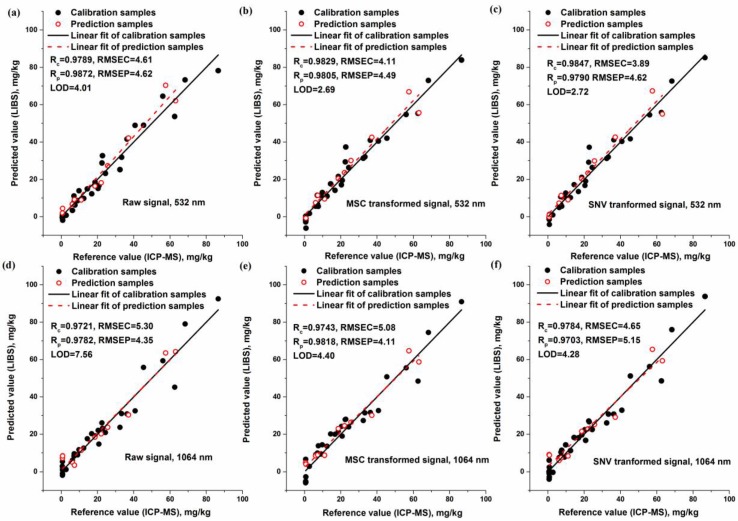
Relationship between reference value and LIBS measured value that calibrated with the variables of (**a**) raw signal of 532 nm excitation; (**b**) MSC transformed signal of 532 nm excitation; (**c**) SNV transformed signal of 532 nm excitation; (**d**) raw signal of 1064 nm excitation; (**e**) MSC transformed signal of 1064 nm excitation; (**f**) SNV transformed signal of 1064 nm excitation. (R_c_: R in calibration set, R_p_: R in prediction set, RMSEC: RMSE in calibration set, RMSEP: RMSE in prediction set).

In general, 532 nm laser pulse obtained better calibration results, with lower RMSEs, LODs and higher values of R. The sensitivity of 532 nm excitation was obviously higher than that from 1064 nm excitation, with a LODs around two times lower. It might be credited to the higher noise induced by 1064 nm excitation. However, the improvement in the aspect of prediction accuracy was limited when using 532 nm excitation. In addition, calibration with the emission from Cr I 425.43 nm showed better overall sensitivity than that from Cr I 427.48 nm. However, the prediction accuracy for both emission lines was similar. Among the preprocessing methods, best calibration results were achieved with the MSC and SNV normalizations. It has been proven that SNV normalization could be used to compensate the laser fluctuation [40]. The improvement of background and area normalizations was limited, and the sensitivity of those was even reduced. Scatter plots that describe the calibration performance of raw signal, MSC normalized signal and SNV normalized signal with 532/1064 nm excitations are shown in Figure 8. 

Apparently, the samples after normalization dispersed closely to the linear fitting line, and the sensitivity was greatly improved. Since SNV normalization could be applied under different experimental conditions, it might be suited for further application. Hence, using the combination of 532 nm excitation and SNV normalized signal of Cr I 425.43 nm was recommended for the detection of chromium content in rice leaves.

## 4. Conclusions

In this study, we investigated the influence of laser wavelength on the signal (sensitivity and stability) and plasma features (electron density and temperature) for the detection of chromium content in rice leaves. We found that the predominant laser wavelength mechanism might differ with the variations of delay time, pulse energy and LTSD. The effect of wavelength on chromium signal might be the result of transition energy, inverse bremsstrahlung and ablation efficiency. With the comparison of the sensitivity and stability, the experimental parameters including delay time, gate width, pulse energy and LTSD were optimized at 4/4 μs, 16/16 μs, 90/80 mJ and 98/99 mm for 532/1064 nm excitations, respectively. In the aspect of quantification capability, 532 nm excitation gave better detection sensitivity than 1064 nm excitation, with a LOD two times lower. However, the improvement of 532 nm excitation in prediction accuracy was limited. The best calibration result was achieved by relating the SNV normalized signal of Cr I 425.43 nm with reference chromium content, with R of 0.9849, RMSE of 3.89 mg/kg, LOD of 2.72 mg/kg for 532 nm excitation and R of 0.9784, RMSE of 4.65 mg/kg, LOD of 4.28 mg/kg for 1064 nm excitation. The proposed approach might provide the first proof-of-principle data for application of LIBS for quantification of chromium content in rice leaves with improved sensitivity and accuracy.

## Figures and Tables

**Table 1:** ICP-MS measured chromium content in rice leaves with five different treatments (range: the range of chromium content, mean ± SD: the mean and standard deviation of chromium content).

Cr Stress Level (µM)	Sample Number	Range (mg/kg)	Mean ± SD (mg/kg)
0	9	0.58–0.80	0.68 ± 0.08
25	11	2.67–12.37	7.84 ± 2.65
50	11	14.35–32.37	21.52 ± 4.87
75	7	22.49–57.63	39.00 ± 10.86
100	5	56.03–86.60	67.32 ± 11.62

**Table 2:** Spectral properties of two emission lines of chromium (*λ_ki_*: transition wavelength, *A_ki_*: transition probability, *E*: energy, i and k: lower and higher levels).

	*λ_ki_* (nm)	*A_ki_* (s^−1^)	*E_i_*–*E_k_* (eV)
Cr (I)	425.43	3.15 × 10^7^	0–2.91
Cr (I)	427.48	2.2 × 10^7^	0–2.90

**Table 3:** Calibration results based on 532/1064 nm excitations with different preprocessing methods.

Wavelength	Variables (nm)	Preprocessing Methods	Calibration				Prediction	
			R	RMSE (mg/kg)	LOD (mg/kg)		R	RMSE (mg/kg)
532 nm	425.43	raw	0.9789	4.61	4.01		0.9872	4.62
		background normalization	0.9780	4.69	4.83		0.9680	6.38
		area normalization	0.9857	3.76	4.53		0.9834	4.40
		SNV transformation	0.9847	3.89	2.72		0.9790	4.62
		MSC transformation	0.9829	4.11	2.69		0.9805	4.49
	427.48	raw	0.9725	5.26	5.34		0.9828	4.75
		background normalization	0.9705	5.46	6.44		0.9697	5.95
		area normalization	0.9845	3.91	6.07		0.9831	4.24
		SNV transformation	0.9852	3.82	3.61		0.9820	4.33
		MSC transformation	0.9848	3.87	3.56		0.9836	4.30
1064 nm	425.43	raw	0.9721	5.30		7.56	0.9782	4.35
		background normalization	0.9715	5.36		9.02	0.9509	6.66
		area normalization	0.9764	4.86		8.40	0.9707	5.04
		SNV transformation	0.9784	4.65		4.28	0.9703	5.15
		MSC transformation	0.9743	5.08		4.40	0.9818	4.11
	427.48	raw	0.9791	4.56		11.35	0.9779	4.38
		background normalization	0.9755	4.95		13.74	0.9722	4.87
		area normalization	0.9864	4.09		12.70	0.9842	3.67
		SNV transformation	0.9823	4.19		6.24	0.9846	3.83
		MSC transformation	0.9704	5.46		6.61	0.9930	2.63
